# ENGAGEMENT OF FAMILY IN ASSISTED LIVING: SECONDARY QUALITATIVE ANALYSIS USING THE ADAPTIVE LEADERSHIP FRAMEWORK

**DOI:** 10.1093/geroni/igad104.1722

**Published:** 2023-12-21

**Authors:** Youngmin Cho, Victoria Crowder, Ayomide Bankole, Lixin Song, Bei Wu, Ruth Anderson, Anna Beeber

**Affiliations:** University of North Carolina at Chapel Hill, Chapel Hill, North Carolina, United States; University of North Carolina at Chapel Hill, Chapel Hill, North Carolina, United States; UNC-Chapel Hill, Chapel Hill, North Carolina, United States; University of Texas Health Science Center at San Antonio, San Antonio, Texas, United States; New York University, New York City, New York, United States; University of North Carolina at Chapel Hill, Chapel Hill, North Carolina, United States; Johns Hopkins University, Baltimore, Maryland, United States

## Abstract

Assisted living (AL) residents are at high risk for safety issues (e.g., falls) and poor quality of life (QoL) because they can lose their personal preferences and autonomy in personal care or social activities once they enter AL. Family engagement (FE) plays a significant role in addressing these challenges while considering residents’ needs and preferences. The purpose of this study was to explore family caregivers’ perspectives about FE strategies in AL and contextual factors influencing FE. This study used a secondary analysis of the interview data from a larger study investigating 34 family caregivers’ opinions on AL safety and strategies to increase FE. The Adaptive Leadership Framework guided our directed content analyses to identify how family caregivers address the challenges with AL residents and staff. Family caregivers described adaptive leadership strategies pertaining to getting the residents to recognize the seriousness of their health status, finding available resources to support them, and planning for potential challenges. Family caregivers also described adaptive and collaborative work (with AL residents or staff) strategies such as advocating for resident needs, involvement in direct care or social activities, shared understanding of care plan, and making decisions together. We also found that contextual factors such as fear of speaking up and retaliation, previous experiences in FE, influenced family caregivers’ engagement in AL safety. These findings highlight the important role of family caregivers in addressing AL resident safety challenges. Also, our study provides the rationale for developing practical FE strategies to enhance residents’ safety and QoL.

